# Rethinking BMI and Obesity Management: The Transformative Role of Artificial Intelligence

**DOI:** 10.7759/cureus.54995

**Published:** 2024-02-26

**Authors:** Abdulqadir J Nashwan, Muad Abdi Hassan, Majd M AlBarakat

**Affiliations:** 1 Nursing Department, Hamad Medical Corporation, Doha, QAT; 2 Medical Education Department, Hamad Medical Corporation, Doha, QAT; 3 Faculty of Medicine, Jordan University of Science and Technology, Irbid, JOR

**Keywords:** body mass index (bmi), artificial intelligence, precision medicine, weight management, obesity

## Abstract

The body mass index (BMI) is a longstanding, simple, and cost-effective tool for classifying individuals by weight, useful for rapidly screening populations for obesity-related health risks. However, its failure to account for muscle mass, bone density, and fat distribution can lead to misclassifications. This editorial explores the transformative potential of artificial intelligence (AI) in redefining or potentially replacing BMI as a tool for enhancing weight management strategies. Furthermore, it illustrates how AI can offer personalized health assessments, predictive analytics, and tailored interventions, overcoming BMI's shortcomings. By analyzing genetic, lifestyle, and medical data, AI enables a more nuanced approach to weight management, signifying a shift toward precision or personalized medicine. AI-driven virtual assistants enhance weight management by offering continuous support, motivation, and reminders, while AI algorithms analyze medical imaging for precise body composition assessment. AI also aids in early metabolic disorder detection and fosters community support among individuals with similar health goals. However, ethical concerns, data privacy, and algorithm biases require careful attention. Collaboration among healthcare professionals, researchers, and tech developers is vital for maximizing AI's positive impact on public health.

## Editorial

The utilization of body mass index (BMI) as a primary metric for assessing obesity and overall health has been a standard practice for decades. BMI, a simple calculation based on height and weight, has provided a quick and easy method for classifying individuals into various weight categories (BMI=weight (kg)/(height (m))^2^ or 703 x weight (lbs)/(height (in))^2^ [1]. However, this method has limitations and controversies, highlighting the need for a more reliable approach to understanding obesity and health.

One of the primary advantages of BMI is its simplicity and cost-effectiveness [1]. It allows for rapidly screening populations, identifying those at potential risk for obesity-related health conditions [1]. Despite its widespread use, BMI overlooks several critical factors, such as muscle mass, bone density, and fat distribution, leading to potential misclassifications [2]. For instance, athletes with high muscle mass may be categorized as overweight or obese despite having low body fat percentages. Conversely, individuals with a normal BMI might carry a higher risk of metabolic diseases if they have a high body fat percentage, particularly in the abdominal area.

These limitations underscore the need for a more comprehensive and personalized approach to measuring and managing obesity - a need that artificial intelligence (AI) is uniquely positioned to fill. AI's advanced analytical capabilities promise to revolutionize healthcare professionals' approach toward these health issues, offering a transformative leap from the traditional BMI metric [3]. AI can create personalized treatment plans considering an individual's unique characteristics, including genetic information, medical history, lifestyle factors, and dietary habits [3]. By analyzing vast datasets, AI develops tailored intervention strategies that are far more effective in achieving desired health outcomes [3]. Additionally, AI's ability to accurately predict the risk of obesity-related complications by analyzing various factors heralds a new era of early intervention and preventive healthcare [3].

In addition, AI-driven virtual assistants can provide continuous support and guidance to individuals on their weight loss journey [4]. These assistants can answer questions, offer motivation, and provide reminders for medication or exercise, enhancing overall patient engagement [4]. AI algorithms can analyze medical imaging data to assess body composition accurately, allowing healthcare professionals to monitor fat distribution and muscle mass changes, enabling a more precise evaluation of treatment effectiveness [4].

AI can also detect metabolic disorders early by analyzing patterns in patient data. Early intervention can prevent the progression of such disorders, reducing the risk of obesity-related complications. Moreover, AI-powered platforms can connect individuals with similar health goals, fostering a sense of community support. Social interaction and shared experiences can enhance motivation and adherence to lifestyle changes [5].

It is essential to note that AI implementation in healthcare requires careful consideration of ethical considerations, data privacy, and potential biases in algorithms. However, collaborating between healthcare professionals, researchers, and technology developers could maximize the positive impact of AI on public health.

In summary, integrating AI into managing BMI and obesity signifies a monumental shift towards precision medicine. By providing personalized assessments, predictive analytics, and tailored interventions, AI is poised to redefine the landscape of weight management. This enhances the efficacy of treatments and empowers individuals in their journey towards achieving optimal health. As we stand on the cusp of this new era, the promise of AI in transforming the fight against obesity and related complications is exciting and inspiring, heralding a future where healthcare is more personalized, proactive, and effective.
